# Wave Function Parity Loss Used to Mitigate Thermal Broadening in Spin-orbit Coupled Zigzag Graphene Analogues

**DOI:** 10.1038/srep40546

**Published:** 2017-01-16

**Authors:** Mohammad Abdullah Sadi, Gengchiau Liang

**Affiliations:** 1 Department of Electrical and Computer Engineering, National University of Singapore, 117576, Singapore

## Abstract

Carrier transport through a graphene zigzag nanoribbon (ZNR) is possible to be blocked by a p-n profile implemented along its transport direction. However, we found that in cases of analogous materials with significant intrinsic spin-orbit coupling (SOC), i.e. silicene and germanene, such a profile on ZNR of these materials allows transmission mostly through spin-orbit coupled energy window due to the loss of the parity of wave functions at different energies caused by SOC. Next, a p-i-n scheme on germanene ZNR is proposed to simultaneously permit edge transmission and decimate bulk transmission. The transmission spectrum is shown to mitigate the effect of thermal broadening on germanene and silicene ZNR based spin-separators by improving spin polarization yield by 400% and 785%, respectively, at 300 K. The importance of proper gate voltage and position for such performance is further elucidated. Finally, the modulation the current output of the proposed U-shape p-i-n device while maintaining its spin polarization is discussed.

Silicene and germanene are honeycomb analogues of graphene which have been successfully synthesized experimentally (silicene on ZrB_2_^1^ and Ir(111)^2^, germanene on SiO_2_/Si^3^ and Au(111)^4^) following their theoretical prediction^5^. Similar to graphene, silicene^6^ and germanene^7^ are predicted to possess Dirac cones, rendering them exceptionally high room-temperature mobilities of ~2.6 × 10^5^ cm^2^ V^−1^ s^−1^ and ~6 × 10^5^ cm^2^ V^−1^ s^−1^, respectively. More importantly, silicene and germanene have larger intrinsic spin-orbit coupling strengths^8^ (λ_SO_, half of spin-orbit coupling (SOC) induced bandgap) of 4.2 meV and 11.8 meV, respectively, compared to that of graphene (~24 μeV)^9^. Although two-dimensional transition metal dichalcogenides^10^ possess even greater SOC, in the order of 100 meV range, these have poorer mobilities in the order of 100 cm^2^ V^−1^ s^−1^. For graphene, rivalling the intrinsic SOC of germanene while maintaining high mobility can be only achieved by proximity effects of transition metal dichalcogenides^11^,^12^, as other approaches such as the introduction of adatoms^13^,^14^ severely hamper mobility despite significantly increasing SOC. However, unlike the proximity effects which has not been explored yet in terms of device applications, there is a vast amount of literature on applications of intrinsic SOC of graphene analogous materials. SOC leads to a plethora of electronic phases of these materials controllable by external electric field^15^, magnetic field^16^ and photo stimulation^17^; manipulations of these phases provides spin-separators^18^,^19^,^20^, spin-filters^8^,^20^,^21^,^22^, and other spin-based logic devices^23^. This indicates silicene and germanene, derived from elements commonly used in the semiconductor industry, may satisfy the craving for non-magnetic spintronic devices in the scientific community^24^,^25^,^26^. However, during device operation at room temperature, thermal broadening of Fermi-distribution will invoke current across several kTs of energy window. This severely reduces the prospect of silicene and germanene to satisfy the craving for non-magnetic spintronic device^24^,^25^,^26^, as unpolarized bulk contribution will occur despite operation close to Dirac point. Various endeavours to increase the strength of spin-orbit coupling in these materials, such as by halogenation^27^, has thus far seen limited success.

Zigzag nanoribbon (ZNR) is the one of the most promising structure for spintronic application of graphene analogous material, as the transmission through 2 × λ_SO_ energy window is mostly edge localized with opposite spins on the edges^28^,^29^ due to quantum spin Hall effect. Very recently, ZNR for graphene has been successfully fabricated where the edge states have been observed^30^. For graphene ZNR, a valley-valve and a valley-filter^31^ were proposed which required judicial use of p-n junctions. Transmission is blocked by the use of p-n junction, for which the explanation of valley-valve effect^32^ was put forward. However, the forbiddance of transmission between the first valence band and the first conduction band in graphene ZNR was later revealed to be part of broader pattern dictated by the contrasting parity properties^33^ of the wave functions of the bands through which the transmission occurs.

In this work, firstly we extend the study to explore the parity property of bands of Group-IV ZNR with significant λ_SO_ by investigating transport through a p-n junction. It is revealed that high transmission occurs in the λ_SO_ window exclusively, a consequence of wave functions’ parity violation in that energy window. Secondly, from the study of the p-n transmission spectrum, a p-i-n potential profile is proposed to be applied on germanene ZNR to produce a transmission spectrum that drastically exclude bulk transmission while letting mostly edge localized transmission through ±λ_SO_ energy window.

The p-i-n scheme can be used to mitigate the effect of thermal broadening of Fermi distribution on spintronic applications of graphene analogue ZNRs, as demonstrated by enhancements of spin polarization (SP) of output current at room temperature from below 0.2 to above 0.6, and from below 0.1 to above 0.6, when implemented on U-shape germanene and silicene spin-separators respectively. The need of high gate voltages and the proper positioning of gates to allow long intrinsic region is also explained in the study. Finally, it is illustrated that the current output of the device can be varied substantially without any significant change of SP of the current output by toggling the source-to-drain voltage and adjustment of p and n regions. Therefore, the implementation of p-i-n scheme is expected to bolster the aspiration of room temperature application of silicene and germanene ZNR based spintronic devices by mitigating the thermal broadening induced spin decay in such devices.

## Results and Discussion

### P-N Transmission in ZNR

**Figure 1:**
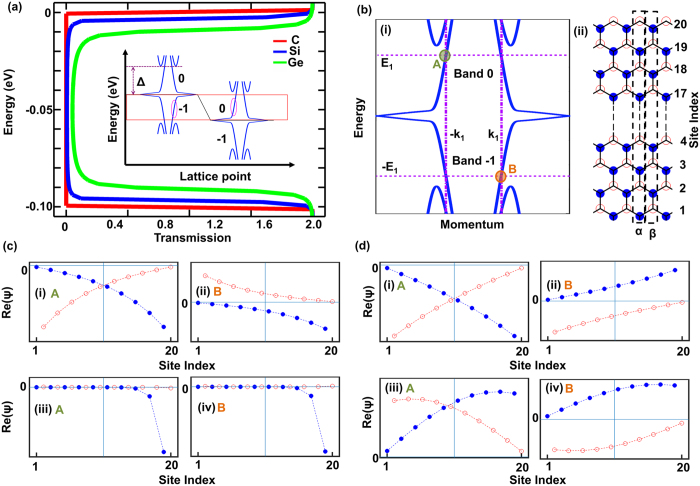
P-N junction transmission for graphene analogue ZNRs showing high transmission in energy windows containing *λ*_SO_ portion of any band (**a**). Inset: schematic of energy-momentum dispersion of the p-n region, with Δ representing the difference between first and second conduction band minima. The band diagram of nanoribbon graphene analogue ZNR marked with foci of wave function investigations is illustrated in (b-i), and the *α* and *β* quasi-lattices as well as site index of the nanoribbon is labelled in (b-ii). The blue filled circles represent one sublattice and the red empty circles represent the other. The real portion of up-spin wave function of the *α* quasi lattice is plotted against site index for the forward moving state of the first conduction band at E = 5 meV (marked as A) and for the first valence band at at E = −5 meV (marked as B) for graphene ZNR in (c-i) and (c-ii) illustrating perfect even and odd parity respectively, and for germanene ZNR in (c-iii) and (c-iv) respectively demonstrating loss of parity. Corresponding cases for the forward moving state of the first conduction band at E = 0.1 eV (marked as A) and for the first valence band at E = −0.1 eV (marked as B) for graphene ZNR in (d-i) and (d-ii) again illustrating perfect even and odd parity respectively, and for germanene ZNR in (d-iii) and (d-iv) illustrating near-even parity and near-odd parity respectively.

Transmission through p-n junction in ZNRs of graphene, silicene, and germanene is illustrated in Fig. 1(a). The potential energy difference at the junction is set to 0.1 eV, which is required to be less than Δ for all the materials, where Δ, as a function of both the type of material and nanoribbon width, is the energy gap between the minima of the first and the second conduction band as depicted in the inset of Fig. 1(a). The transmission occurs through the first valence band (marked as −1) and the first conduction band (marked as 0) of the ZNRs for the energy window 0 to −0.1 eV. There is virtually no transmission for graphene ZNR in this energy window, as illustrated in Fig. 1(a). This is expected as the wave function of the ZNR of a single quasi-lattice is an even function for band 0 and an odd function for band −1, and transmission is forbidden between bands whose wave functions have the opposite parity^33^ when a sharp junction is present. The parity property of wave function arises from the inversion symmetry of the zigzag nanoribbon across the width when it has even number of chains.

In the cases of silicene and germanene ZNRs with even number of chains, as shown in Fig. 1(a), high transmission through the p-n junction occurs for an energy window corresponding to the *λ*_SO_ of each material. The transmission decays outside the p region’s nanoribbon *λ*_SO_ and width modulated (due to crosstalk; see Fig. S1) energy window rapidly for half the potential energy difference between intrinsic and n region. Then, the transmission picks up gradually and becomes high again for n region’s *λ*_SO_ and width modulated window. To understand the p-n transmission spectrum of ZNRs with significant *λ*_SO_, we investigate wave functions across one-quasi lattice for band 0 and band −1 for graphene and germanene at point A and B as depicted in Fig. 1(b-i). The loci of wave function investigations, namely A and B, are the intersections of the energy E_1_ and −E_1_ respectively with the forward moving states of band 0 and band −1, respectively. The repeating unit of the nanoribbon can be divided into *α* and *β* quasi-lattices, which are illustrated by Fig. 1(b-ii). The site index, furthermore, indicates the width of the nanoribbon under investigation to be 20 atoms across each quasi-lattice. However, for our analysis, only wave function distribution across *α* quasi-lattice is sufficient, as the conclusions are applicable for *β* quasi-lattice as well. Similarly, only the wave functions for the up-spin bands is considered in the following investigations, though the implications will remain same for the down-spin bands.

For E_1_ = 5 meV, i.e. when E_1_ and −E_1_ are inside the *λ*_SO_ window for germanene, the wave function of the graphene ZNR at A is perfectly even across the middle of the nanoribbon, and perfectly odd at B, as shown in Fig. 1(c-i) and (c-ii), respectively. The blue markers and red markers are used for showing wave functions of different sublattices for clarity; however, each perfect parity is achieved for the complete wave function. Unlike graphene ZNR, the wave functions at A and B for germanene for the same case lose the parity information, as shown in Fig. 1(c-iii) and (c-iv), respectively. To maintain the parity of wave function, the magnitude of its distribution must be symmetrical about the middle of the nanoribbon. The spin-orbit coupling in germanene induces spin-polarized edge states in the ±*λ*_SO_ window; the skew of wave function distribution towards one edge for one particular spin for a particular direction of propagation will result in loss of its parity property. Away from the ±*λ*_SO_ window for germanene, for E_1_ = 0.1 eV, unsurprisingly, the wave function parity for graphene ZNR is still maintained at A and B, as shown in Fig. 1(d-i) and (d-ii). However, for germanene ZNR, there is significant change between the wave functions for E_1_ = 5 meV and those for E_1_ = 0.1 eV. It is delineated by the snapshots in Fig. 1(d-iii) and (d-iv) that if we investigate continuously away from the ±*λ*_SO_ energy region we will find that the wave function of germanene ZNR at A gradually approaches even parity, and that of B gradually approaches odd parity as the wave function distribution becomes delocalised, although unlike those of graphene ZNR, these do not possess perfect parity. This phenomenon holds for all graphene analogues with significant SOC, such as silicene and stanene.

The transmission through the p-n junction in the energy window 0 to −0.1 eV arises from the parity properties of the forward moving valence band in the p region and the forward moving conduction band in the n region. From the wave functions of germanene ZNR discussed above, it follows that within the energy window between 0 and *λ*_SO_, the forward moving valence band has strongly localized, parity-lost wave functions while the forward moving conduction band has wave functions strongly recovered towards having even parity. Similarly, one of the forward moving bands has parity-lost wave functions and the other has those with strong recovery of parity property at the energy window from −0.1 eV+ *λ*_SO_ to −0.1 eV. In both of these cases, presence of one band with parity-lost wave functions corresponds to high transmission in both these energy windows. The minimum of the transmission, after a gradual decay, occurs at the middle of the energy window between 0 eV to −0.1 eV, which is correlated with the forward moving states of the valence band and the conduction band recovering towards odd and even parity, respectively. Therefore, for effective blocking of transmission, both the bands need to possess strong but opposite parity property, which in case of graphene ZNR is satisfied for all energies in the window from 0 eV to −0.1 eV, but not for its analogues with significant *λ*_SO._

### P-I-N Transmission in ZNR

**Figure 2:**
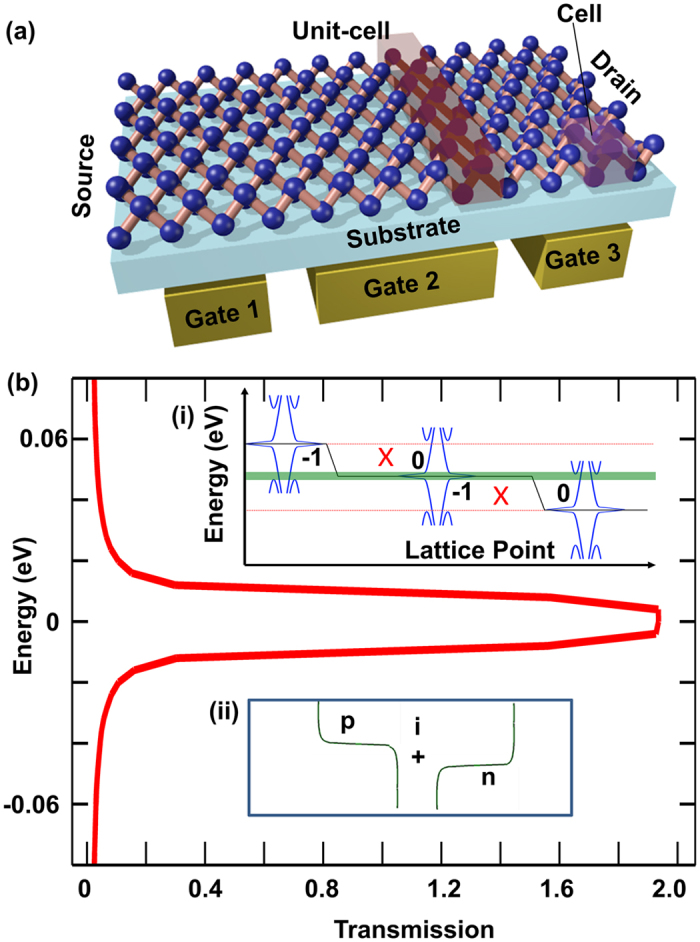
P-I-N ZNR device structure with the three gates between the source and drain. The 4 atom cell is boxed in purple (color online), and the unit-cell is boxed in red (**a**). P-I-N device scheme yielding high isolated transmission in spin-orbit coupled window for 10 cell wide ZNR (**b**). Gate voltages of +Δ/q, 0 V, and −Δ/q are used for p, i, and n region respectively. Insets: (i) The role of the band alignments of ZNR that gives rise to the p-i-n transmission, and (ii) an illustration of how p-i and i-n transmissions work together to achieve it.

A p-i-n scheme is implemented on germanene ZNR via three gates distributed between a source and a drain, as shown in Fig. 2(a). The device scheme is designed to isolate the edge polarized transmission through ±*λ*_SO_ energy and suppress the bulk transmission. The gate voltages applied at the p and n region, which correspond to Gate 1 and Gate 3, respectively, have the magnitude of Δ/q, which is 0.21 V for the 10 cell wide germanene ZNR. A long intrinsic region of 50 unit-cell under Gate 2 is maintained to validate band diagram approximations. Both cell and unit-cell are delineated in Fig. 2(a). The p and n regions are extended into infinity. The gate voltages are set to ensure the maximum decay of bulk transmission, as the minimum transmission will be smaller with the larger junction potential difference due to the gradual nature of the transmission decay as shown in Fig. 1(a). A gate voltage larger than Δ/q will introduce additional transmission from the new band introduced in transport window, which has the same parity instead of opposite parity of the band on the other side of the junction.

When such a p-i-n profile is applied on germanene ZNR, isolated high transmission is achieved for width modulated 2 × *λ*_SO_ energy window, as illustrated by Fig. 2(b). The crosses in the energy dispersion diagram in Fig. 2(b-i) depicts that the upper part of the transmission is cut off by the p-i junction, while its lower part is cut off by the i-n junction as electrons pass through band 0 and −1, unless passing through *λ*_SO_ portion of a band at that junction. The p-i-n transmission spectrum can be envisioned, as shown in Fig. 2(b-ii), as a juxtaposition of two p-n transmission spectra, whose nature is shown in Fig. 1(a). This synthesized p-i-n transmission profile is expected to combat the effect of Fermi function broadening at high temperature on spintronic device performance by promoting edge localized transport while suppressing that of bulk. The effectiveness of the profile can be verified through a spintronic device based on germanene ZNR.

### P-I-N Enhanced Performance of ZNR based Spintronic U-shape Device

To evaluate the effectiveness of the p-i-n profile in achieving mitigation of thermal broadening induced spin decay, it is implemented on ZNR based germanene U-shaped device. The U-shape device collect edge localized opposite spins (aligned perpendicular to the device) at its two different drains^20^. The lengths of the three arms of this structure are set to 30 unit-cells each, and the width of Arm-P, Arm-Q, and Arm-R are set to 10, 4, and 4 cells, respectively. The p region at the source and n region at the drains are treated as infinite. For each arm the transition region is kept short at 2 unit-cell to approximate step like potential, and the intrinsic region is 25 unit-cell long. The source-to-drain voltage (V_DS_) is 2 × λ_SO_/q unless otherwise mentioned. Ballistic transport model in the channel is adopted in the study, and the effect of phonons is assumed to be negligible since according to a previous study by Bishnoi *et al*. germanene is predicted to have phonon induced spin dephasing length over 1.5 μm in the temperature range 0–300 K^34^, which is several tens of times longer than the length of this device. The intrinsic regions have flat potential of 0 eV maintained by a gate. The potential energy difference in both p-i and i-n junctions is maintained at Δ eV of Arm-P, by adjusting the other two gates.

**Figure 3:**
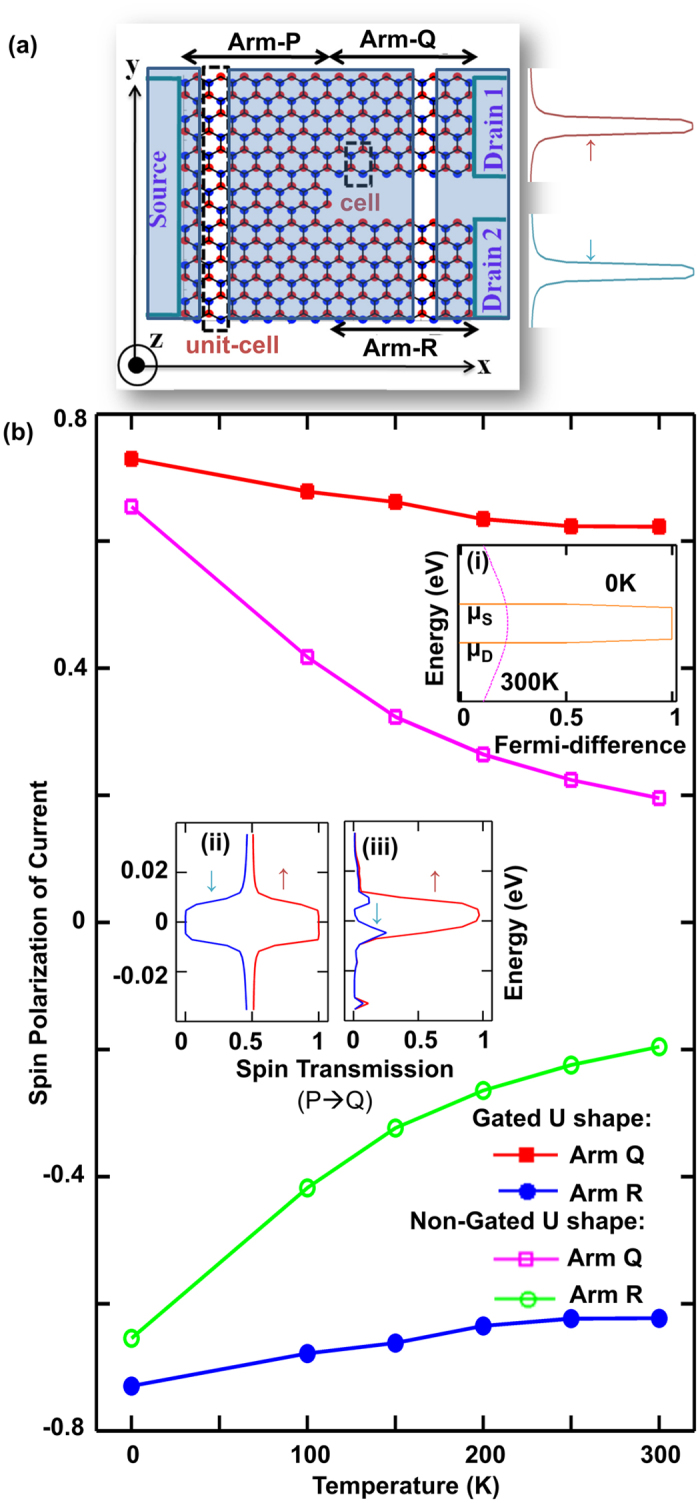
U-shape device structure, consisting of one source and two drains (**a**). The translucent blocks represent the gates on the device. The red and blue atoms are at different sublattices. The p-i-n transmission derived from nanoribbon separate into opposite spin transmissions at the drains as illustrated at right. (**b**) Spin polarization of current for p-i-n gated U-shape device contrasted against non-gated U-shape device. Insets: Despite thermal broadening of Fermi distribution difference between source and drain (i) spin polarization decay due to it is largely mitigated for p-i-n U-shape device as the equilibrium spin transmission (Red arrow representing up-spin and blue arrow representing down-spin along z-axis) from Arm-P to Arm-Q for non-gated U-shape device (ii) contrasted with that of gated U-shape device (iii) shows that the unpolarized transmission is cut off for the p-i-n device.

As illustrated in Fig. 3(b), at room temperature, spin polarization of Arm-Q (SP_Q_) is maintained at 0.623 for p-i-n U-shape device but falls to 0.196 for the non-gated device. The high SP_Q_ is maintained in spite of the vastly widened relevant energy window of transport, as illustrated in Fig. 3(b-i), due to the thermal broadening of Fermi distribution at 300 K. The success of the scheme as mentioned above is evidently owing to the curtailing of transmission outside width modulated 2 × λ_SO_ energy window, as delineated by the contrasting equilibrium spin transmission for Arm-Q of the non-gated U- shape device in Fig. 3(b-ii) and gated U-shape device in Fig. 3(b-iii). The small spike of undesirable spin transmission at the lower half of the spectrum in Fig. 3(b-iii) can be attributed to the geometry mismatch between Arm-P and Arm-Q, as the junction at Arm-Q is responsible for the bottom half of the transmission spectrum. It is unambiguous that the p-i-n configuration is indeed highly effective in preserving SP_Q_ against the detrimental effects of thermal broadening on the performance of germanene ZNR based spintronic device. (Similarly, for silicene U-shape device, an improvement of SP_Q_ from 0.07 to 0.62 at 300 K can be obtained as shown in Fig. S5).

**Figure 4:**
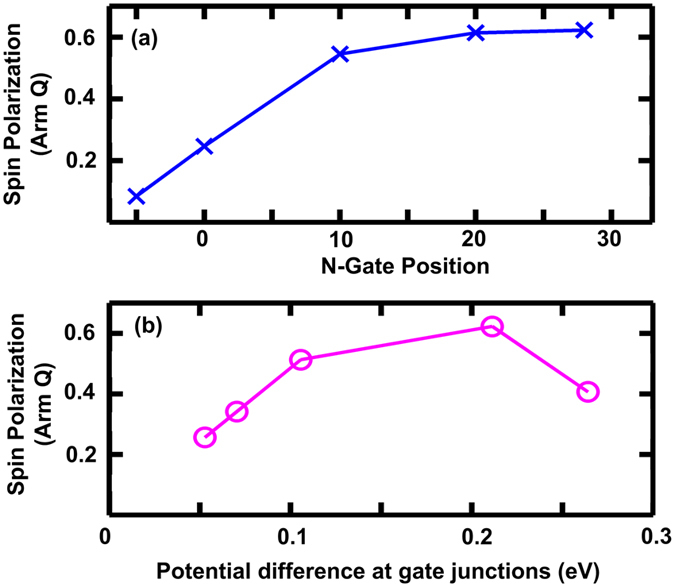
Spin polarization of current in Arm-Q at 300 K (**a**) for varied starting position of n region relative to juncture between Arm-P and Arm-Q illustrating the need for long intrinsic regions and (**b**) for varied potential difference at the p-i (and i-n) junction.

The success of the p-i-n scheme in effectively mitigating thermal broadening depends on two key gate parameters, namely gate positioning and the potential difference across the junctions. A study of the position of the n-gate on the U-shape device as illustrated in Fig. 4(a) reveals that the closer to drain contact the n-gate is deployed the better is the SP_Q_. As the gate is moved further towards the drain, SP_Q_ improves owing of the increase of the length of intrinsic region. This supports the previous assertion that long intrinsic region is crucial to device performance, with the performance saturating for this U-shape device after the intrinsic length of 20 unit-cells of the Arm-Q according to Fig. 4(a). If the n-gate is placed inside Arm-P, the SP_Q_ deteriorates severely, which indicates that the edge localization of the spin polarized current is rapidly lost in the n region as the electrons flow through non-localized bands.

We also demonstrate through Fig. 4(b) that the higher the potential energy difference at each junction, the better is the device performance, albeit for this trend to hold the potential difference should not exceed Δ of Arm-P. As the potential difference at the junctions is increased by altering p and n gate voltages, the decay of transmission outside 2 × *λ*_SO_ energy is enhanced, resulting in smaller unpolarized transmission incorporated into transport and thus higher SP_Q_. The fall of SP_Q_ as this difference exceeds Δ of Arm-P can be attributed to the introduction of new band with similar parity tendency rather than contrasting parity tendency across each junction. For this particular spintronic device, the additional unpolarized current is only introduced at the p-i junction, as Δ of Arm-Q is much larger than Arm-P due to the smaller width of Arm-Q, where i-n junction is located, and the same potential energy difference is maintained across both junctions.

**Figure 5:**
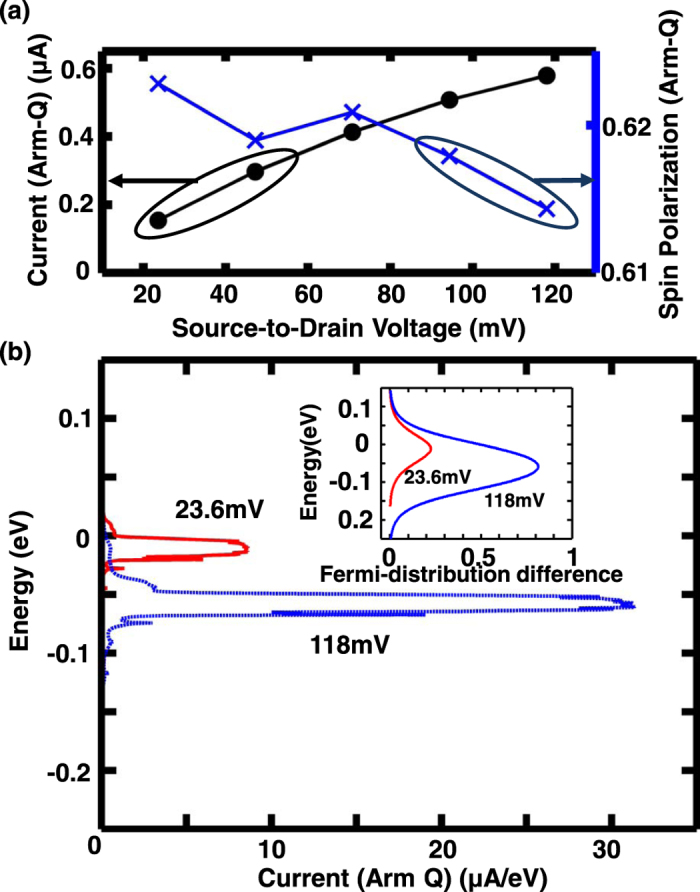
Current voltage characteristics at 300 K explored through (**a**) Arm-Q current (black circles) and SP_Q_ (blue crosses) plots against source-to-drain voltage (V_DS_) ranging from 2 λ_SO_/q to 10 λ_SO_/q and (**b**) plots of current spectra for Arm-Q for V_DS_ of 2 λ_SO_/q (red) and 10 λ_SO_/q (blue), with the inset showing how the increased Fermi distribution difference between source and drain for V_DS_ of 10 λ_SO_/q (blue) compared to that of 2 λ_SO_/q (red) contributes to the increase in current for increased voltage.

Next, we demonstrate that the current output of this proposed structure at 300 K can be modulated without inducing significant decay in SP_Q_. This can be achieved by changing the V_DS_, while maintaining constant potential energy difference of Δ eV at the junctions through adjustment of p and n gate voltages to prevent any detrimental effect of non-optimal potential difference as per discussion beforehand. With this technique in place, increasing V_DS_ from 2 × *λ*_SO_/q to 10 × *λ*_SO_/q increases the Arm-Q current from 0.154 μA to 0.579 μA, while the SP_Q_ resides in the range of 0.614 to 0.623, as shown in Fig. 5(a). SP_Q_ is largely unaffected as the increase in current with increased V_DS_ comes not from an increase in transmission energy window, but from the increase of the magnitude of Fermi distribution difference between source and drain at the spin polarized energy window as illustrated in the inset of Fig. 5(b). The dominant current contribution of spin-polarized window is outlined by the current spectra of Arm-Q plotted for the two V_DS_ in Fig. 5(b). The achievable spin polarized current density is quite significant because the width of Arm-Q is only around 4.5 nm.

Lastly, we would like to note that although our work does not incorporate Hubbard model for electron-electron interaction, we expect our findings to be qualitatively applicable even if the electron-electron interaction effect is significantly stronger than the effects of spin-orbit coupling. Firstly, the electron-electron interactions are not expected to significantly affect band regions with strong parity, i.e. non-localized states as Hosein *et al*.^35^ show that in graphene zigzag nanoribbon, negative differential resistance arising from the parity selective transport involving first valence band and first conduction band is especially robust even when Hubbard U model is applied. Secondly, an in tandem study^36^ of Hubbard model and spin-orbit coupling on graphene zigzag nanoribbon reveals that in case electron-electron interaction is overwhelmingly stronger than spin-orbit coupling there will be a bandgap induced, as well as ferromagnetic^37^ edge states. The bandgap will not affect the spin-polarization of the current output. Both Hubbard model and spin-orbit coupling are expected to result in spin-separation at the edges during transport. In the case of spin-orbit coupling being more prominent, in equilibrium there will be no spin separation, but for forward transport up-spin will propagate along the upper-edge and the down-spin will propagate along the lower-edge of the zigzag nanoribbon. In the case of electron-electron interaction being more prominent, up-spin will congregate along the upper-edge and the down-spin along the lower-edge regardless of transport direction. The edge states induced by electron-electron interaction will again lack wave function parity. Thus the p-i-n scheme for zigzag nanoribbon of graphene analogue material is expected to allow spin-polarized edge localized transmission when the edge states are involved in the transport while blocking the delocalised states, and the sign of spin polarization of electrons for a particular edge is expected to be same both for electron-electron interaction induced edge state and spin-orbit coupling induced edge state for the forward direction of transport.

## Conclusions

In summary, our study delves into the parity property of wave functions for ZNRs of Group-IV materials and reveals its violation for λ_SO_ energy window corresponding to selective high transmission through p-n junction in the ZNR. The p-n transmission spectrum for germanene ZNR nanoribbon is successfully utilized to craft isolated high transmission for spin-orbit coupled energy window using p-i-n scheme. The spintronic application of the isolated transmission is illustrated by germanene U-shape device, whose output SP_Q_ is enhanced by over 400% at room temperature to reach over 60% by applying the p-i-n scheme. Similarly, silicene U-shape device has a performance boost of 785%. Such scheme, moreover, allows for current modulation using V_DS_ without spin polarization decay. Additionally, the importance of high gate voltage and correct placement of gates for effectiveness of such a scheme is elucidated. The research exhibits the effect of λ_SO_ on parity property of graphene analogue ZNRs, and advances the possibility of room temperature spintronic applications of silicene and germanene ZNR based devices.

## Method

To investigate the transport, non-equilibrium Green’s function formalism^38^ is used in this study. The four-band tight binding model Hamiltonian for the graphene analogue materials is adopted from Tsai *et al*.^8^,


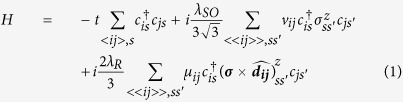


where *t* is the nearest-neighbour hopping term on the honeycomb lattice, 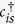 represents the creation operator for the spin-polarization *s* at site *i*, and the intrinsic (*λ*_SO_) and Rashba (*λ*_R_) spin-orbit interactions are represented in the second and the third term respectively. The hopping term for graphene is extracted from Castro Neto *et al*.^39^. The *λ*_SO_ and *λ*_R_ of graphene is assumed to be negligible. Table 1 summarizes the material parameters used in this investigation.

Green’s function is calculated using full inversion to obtain the transmission values. Eq. (2) is used to calculate the current and Eq. (3) the spin polarization of current at each arm (Arm i),


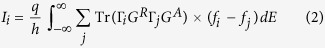



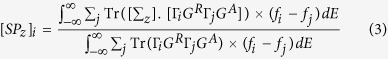


where *f*_*i*_ is the Fermi distribution of the contact at Arm i, and *f*_*j*_ is that of the contact for any Arm j for which *f*_*i*_ ≠ *f*_*j*_. Γ_*i*_ and Γ_*j*_ are the broadening matrices for Arm i and Arm j contact, respectively, Σ_*z*_ contain the z-Pauli matrices as its diagonal terms for summing up and down spin current, and *G*^*R*^ and *G*^*A*^ are the retarded and advanced Green’s functions for Arm i. Surface-green function is computed iteratively by implementing the Sancho-algorithm^40^. In order to cover the energy range in which electrons may contribute significantly to the transport behaviour the integration limits are numerically set to *μ*_*S*_ + 5.5*K*_*B*_*T* and *μ*_*D*_ − 5.5*K*_*B*_*T*, where *μ*_*S*_ and *μ*_*D*_ are electro-chemical potentials at the source and drain terminals, T is the temperature and K_B_ is the Boltzmann constant.

## Additional Information

**How to cite this article**: Sadi, M. A. and Liang, G. Wave Function Parity Loss Used to Mitigate Thermal Broadening in Spin-orbit Coupled Zigzag Graphene Analogues. *Sci. Rep.* **7**, 40546; doi: 10.1038/srep40546 (2017).

**Publisher's note:** Springer Nature remains neutral with regard to jurisdictional claims in published maps and institutional affiliations.

## Supplementary Material

Supplementary Information

## Figures and Tables

**Table 1:** Model parameters of lattice constant *a*, buckling distance Δ_c_, nearest-neighbour hopping parameter *t*, intrinsic spin-orbit coupling *λ*_SO_, and Rashba spin-orbit coupling *λ*_R_ for different materials.

	*a* (Å)	Δ_c_ (Å)	*t* (eV)	*λ*_SO_ (meV)	*λ*_R_ (meV)
Graphene	1.42	0	2.8	0	0
Silicene	3.87	0.44	1.04	4.2	8.66
Germanene	4.06	0.68	0.97	11.8	2.81
