# Supplementary material for: Wave Function Parity Loss Used to Mitigate Thermal Broadening in Spin-orbit Coupled Zigzag Graphene Analogues
Source: Sci Rep. 2017 Jan 16;7:40546. doi: 10.1038/srep40546 (PMC5238368; doi:10.1038/srep40546)
Supplement: Supplementary Information [file srep40546-s1.pdf]

Wave Function Parity Loss Used to Mitigate Thermal Broadening in Spin-orbit Coupled Zigzag Graphene Analogues

Mohammad A. Sadi and Gengchiau Liang

Supplementary Information:

A. The effect of device width on spin polarization of output current

The energy window of high transmission through the p-n junction increases with the width of the nanoribbon of germanene, as shown in Figure S1. The wave functions in the λ_{SO} window adjacent to the dirac point is known to be edge localized. The skewness of the wave function squared (Ψ^2) of up-spin forward moving first conduction band of germanene nanoribbon plotted against energy illustrates that the skewness (barring few anomalies) and thus edge localization is stronger with wider nanoribbon for the range of width investigated in Figure S2, as greater skewness will correspond to greater localization given that it is known that the wavefunctions are localized. The skewness is calculated using the formula, where y= Ψ^2 and $\bar{y}$ is the mean of y:

$$Skewness = \frac{\frac{1}{n}\sum_{i=1}^{n}(y_i - \bar{y})^3}{\left(\sqrt{\frac{1}{n}\sum_{i=1}^{n}(y_i - \bar{y})^2}\right)^3}$$

The weaker localization of wavefunction for smaller width of nanoribbon is the exhibition of cross-talk between edge states. Weaker localization will result in faster recovery towards parity of wavefunction, and thus smaller high transmission window for transport through p-n junction of a narrower nanoribbon. For the p-i-n U-shape device, having a smaller high transmission energy window will imply decreased spin polarized current into the drains from the λ_{SO} window. If the width of Arm-P,

and gate voltages at p and n regions are kept constant, smaller width of Arm-Q results in smaller spin polarization output, as shown in Figure S3.

However, the value of Δ is reduced for wider nanoribbon, reducing the maximum gate potential applicable in p and n regions. This would increase unpolarized transmission for wider nanoribbon as the larger the gate voltage is applied, if no new band is introduced in relevant transport window, the smaller is the unpolarized transmission contribution as shown in Figure 4(b) of the main text. The concerted contributions of increased spin polarized transmission owing to reduced cross-talk and increased depolarized transmission due to reduced gate voltage result in fluctuations of spin polarization of output current in the range of 62% to 67% as the width of Arm-P is increased from 10 to 16 cells as shown in Figure S4, when at p and n regions the gate voltages of magnitude Δ/q of Arm-P is applied on each device.

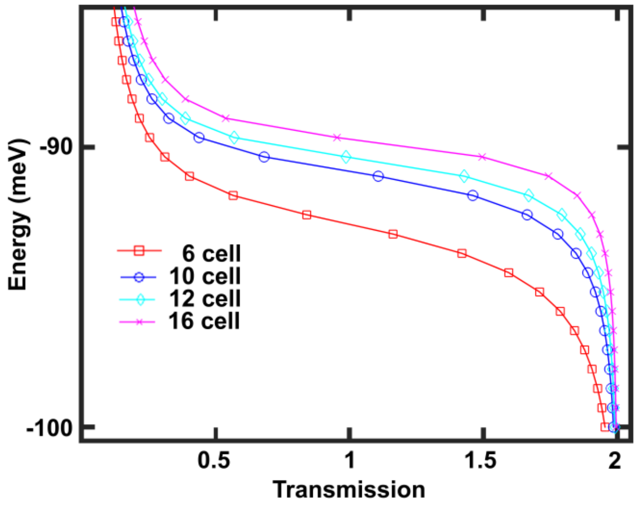

Figure S1. The p-n transmission for germanene nanoribbons with different widths showing increasing energy window of high transmission with width. A gate voltage of 0.1 V is applied at the n region for all the nanoribbons.

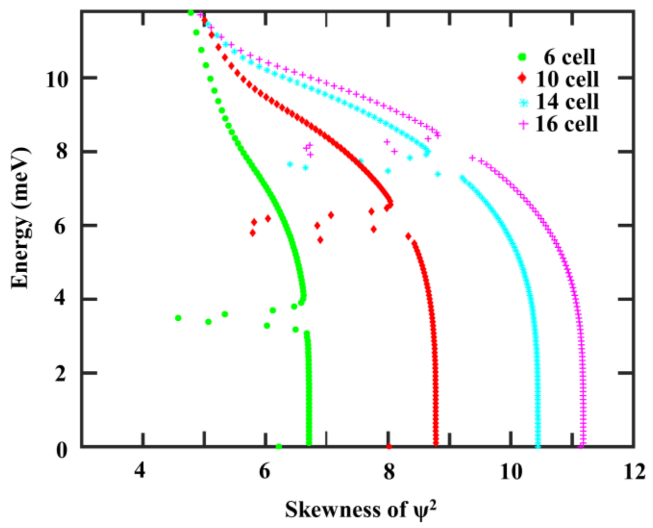

Figure S2. Skewness of the wave function squared for the forward moving up-spin first conduction band of germanene nanoribbons of different widths illustrating that the wave function is more localized for wider nanoribbon in the λ_{SO} energy window, for the range of width explored.

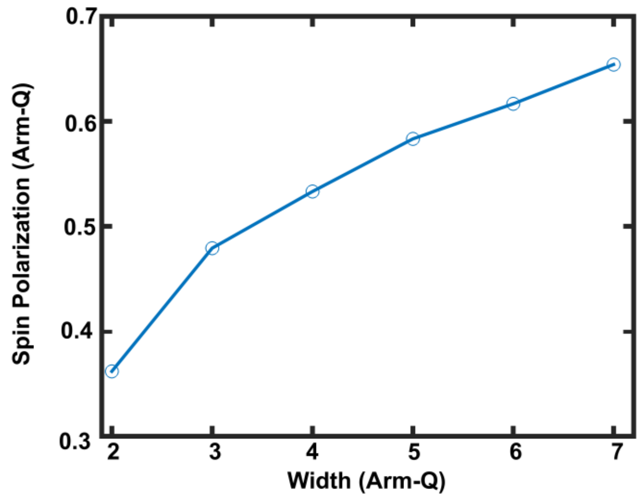

Figure S3. Spin Polarization of Arm-Q of p-i-n U-shape device at 300 K plotted for different widths of Arm-Q, with width of Arm-P set to 16 cells. The magnitude of potential difference at p-i and i-n regions is set to Δ/q of Arm-P. The widths of the drain arms are equal.

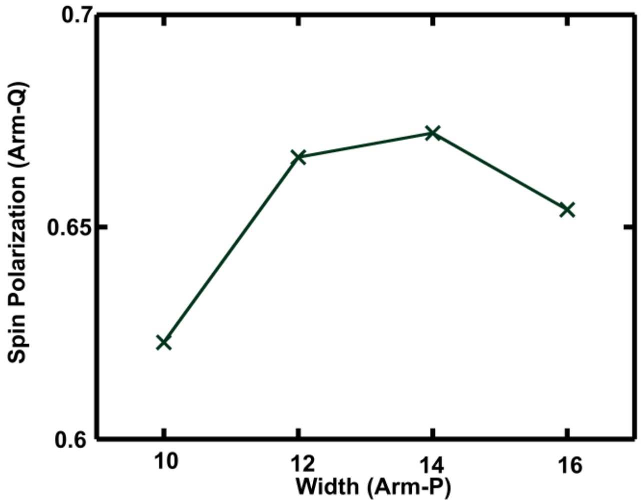

Figure S4. Spin Polarization of Arm-Q of p-i-n U-shape device at 300 K plotted for different widths of Arm-P (nWp), with width of the drain arms of the U-shape device set to nWp/2-1. The magnitude of potential difference at p-i and i-n regions is set to Δ/q of Arm-P.

B. The mitigation of thermal broadening for silicene and stanene spintronic U-shape device

The p-i-n scheme is applicable to silicene and stanene zigzag nanoribbon based spintronic devices as well as those of germanene. However, the implementation of p-i-n scheme across ZNR of a different material requires adjusting the gate potential differences and V_{DS} to the ZNR material's Δ and 2 λ_{SO} respectively. When done such, it is shown in Figure S5 (a) that at 300 K, there is a 785% increase in spin polarization with p-i-n scheme implementation in silicene U-shape spin separator, where the spin polarization yield jumps from 0.07 to 0.62. For stanene, Figure S5 (b) shows a comparatively modest improvement of 28% as the spin polarization climbs from 0.53 to 0.68 at room temperature with the application of the p-i-n profile. The difference between the spin polarization yields of different material based devices under the same scheme arises from the difference in ratio of highly edge-localized transmission coming from ± λ_{SO} energy window, and non-localized transmission coming from outside the window. Greater λ_{SO} results in larger energy window of localized transmission; however, this also results in larger non-localized transmission contribution as the decay of transmission is less significant outside λ_{SO} window, and the maximum gate voltages applicable as limited by Δ of the nanoribbon decreases.

It is to be noted that in demonstrating the mitigation of the impacts of themal broadening, V_{DS} of 2 λ_{SO}/q is used. It is possible to get a spin polarization of almost 1 at 0 K for silicene, germanene, and stanene U-shape device with the same dimensions as in Figure 3 in the manuscript with a V_{DS} of 1 mV applied, due to operation in very strongly edge-localized part of the band. However, at ≥100 K the difference between spin polarization yields of a U-shape device with the aforementioned dimensions with V_{DS} 1 mV and 2λ_{SO}/q is negligible for silicene and germanene. But with larger V_{DS}, current of greater magnitude can be drawn, hence the justification for using V_{DS} of 2 λ_{SO}/q for the U-shape devices.

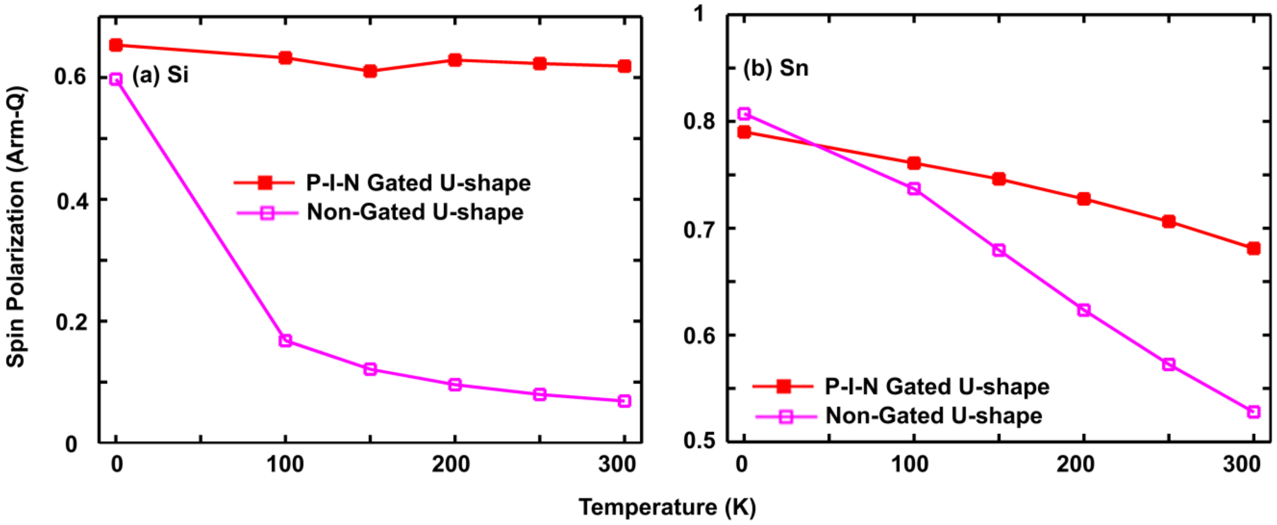

Figure S5. Spin Polarizations of Arm-Q of p-i-n gated U-shape device and non-gated U-shape device with same dimensions as in Figure 3 of the main manuscript are plotted for silicene (a) and stanene (b) against temperature. The magnitude of potential difference at p-i and i-n regions is set to Δ/q of Arm-P, and a V_{DS} of 2 λ_{SO}/q is applied, both Δ and λ_{SO} being material dependent.

C. Marginal loss of wave function parity by Rashba spin-orbit coupling

The parity loss of wave function in germanene ZNR occurs mainly due to intrinsic spin-orbit coupling (λ_{SO}) induced quantum spin Hall effect. However, in the wave function and transport calculations the Rashba spin orbit coupling (λ_R) property of the material is also considered, which breaks the z→-z mirror symmetry. To elucidate the effect of λ_R on the parity property, the λ_{SO} is set to zero, and the real portion of wave function is plotted across site index for the α quasi lattice for forward moving up-spin state of the valence band for germanene ZNR at energy of -0.1 eV in Figure S6 (a) and at energy of -5 meV in Figure S6 (b). At both energies, insignificant deviation from perfect odd parity is observed in presence of only λ_R. Thus the deviation in parity of wave function in Figure 1 in the main manuscript can be concluded to arise mainly from λ_{SO}. However, λ_R does indeed cause deviation from perfect parity of the wave function, albeit the effect is much smaller than that caused by λ_{SO}. The small deviation from perfect odd parity of the wave function can be clearly observed in Figure S6 (c) and (d) where the λ_R coefficient in germanene ZNR is amplified by a factor of ten while λ_{SO} is maintained at zero.

In terms of device performance of p-i-n gated U-shape germanene device, the role of λ_R is marginal due to its minimal influence on the parity of wave function. This is evident from Figure S7, which shows spin polarization output of the device at 300 K is virtually same for zero λ_R, original λ_R, and amplified λ_R, while λ_{SO} is maintained at 11.8 meV.

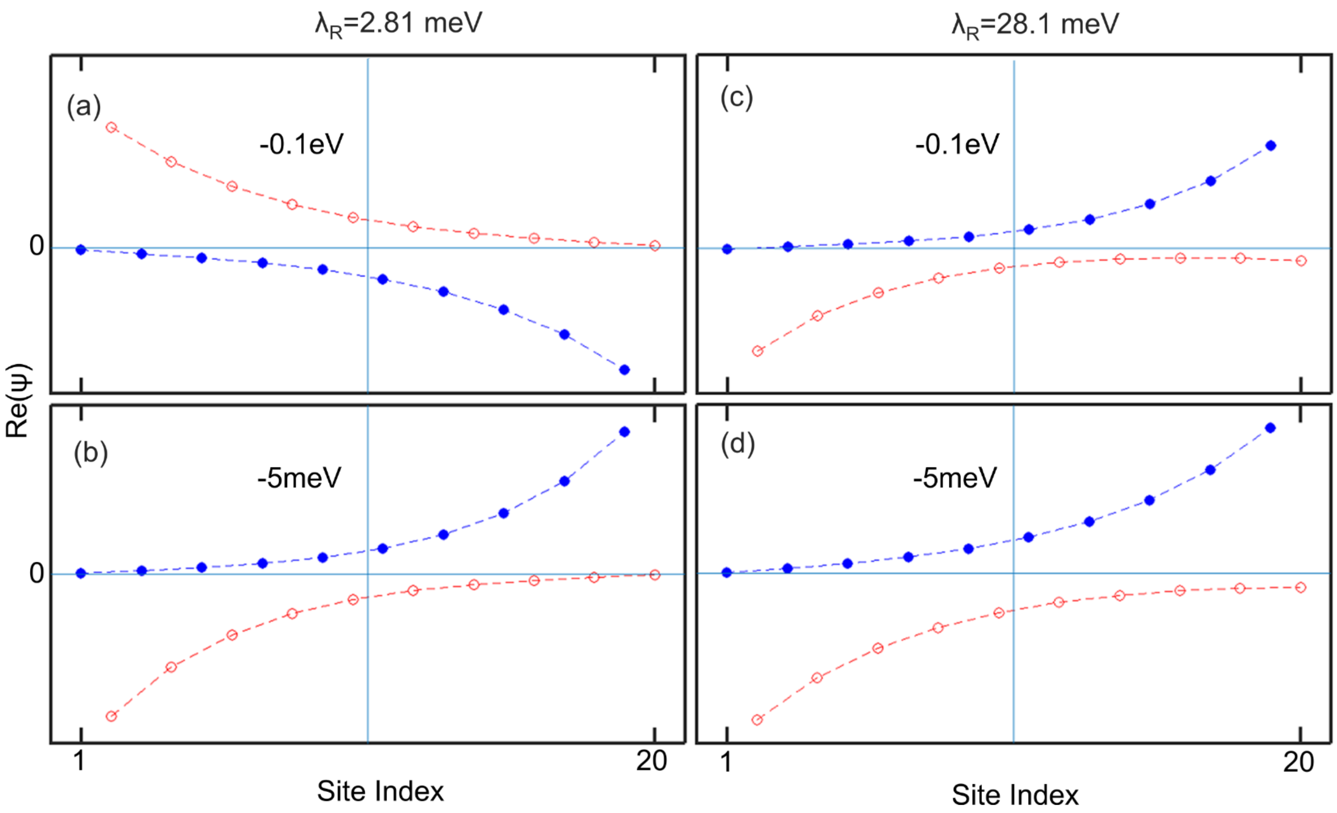

Figure S6. Intrinsic spin-orbit coupling (λ_{SO}) of germanene ZNR is set to zero to investigate the effect of Rashba spin-orbit coupling (λ_R) on the wave function parity property of germanene ZNR. The real portion of up-spin wave function of the α quasi lattice is plotted against site index for the forward moving state for the first valence band at E=-0.1 eV of germanene ZNR in (a) and at E=-5 meV at (b) respectively for the original λ_R=2.81 meV, and in (c) and (d) respectively for amplified λ_R=28.1 meV shows that the parity violation induced by Rashba spin-orbit coupling is minor compared to that induced by intrinsic spin-orbit coupling.

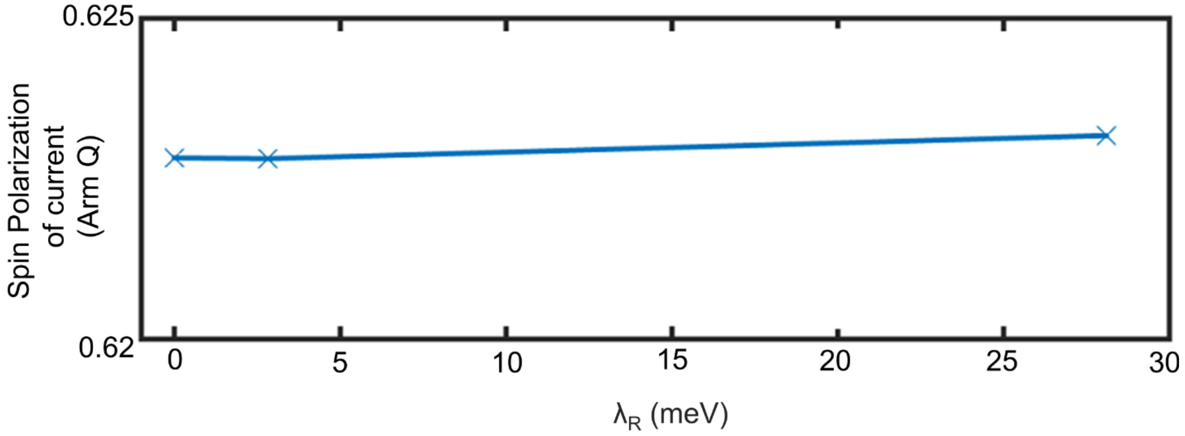

Figure S7. Spin polarization of current at 300K in Arm-Q of p-i-n gated germanene U-shape device with same dimensions as in Figure 3 of the main manuscript showing marginal change with artificial variation of Rashba spin-orbit coupling (λ_R).
